# Green biosynthesis, characterization, and cytotoxic effect of magnetic iron nanoparticles using Brassica Oleracea var capitata sub var rubra (red cabbage) aqueous peel extract

**DOI:** 10.3906/kim-2102-2

**Published:** 2021-08-27

**Authors:** Ömer ERDOĞAN, Salih PAŞA, Gülen Melike DEMIRBOLAT, Özge ÇEVİK

**Affiliations:** 1 Department of Biochemistry, Faculty of Medicine, Aydın Adnan Menderes University, Aydın Turkey; 2 Department of Science, Faculty of Education, Afyon Kocatepe University, Afyon Turkey; 3 Department of Pharmaceutical Technology, Faculty of Pharmacy, Biruni University, İstanbul Turkey

**Keywords:** Green synthesis, iron oxide nanoparticles, red cabbage, *Brassica oleracea var. capitata sub.var. rubra*, breast cancer

## Abstract

The green method of nanoparticle synthesis, which is an environment and living-friendly method, is an updated subject that has appeared as an alternative to conventional methods such as physical and chemical synthesis. In this presented study, the green synthesis of magnetic iron oxide nanoparticles (IONPs) from iron (III) chloride by using *Brassica oleracea var. capitata sub.var. rubra* aqueous peel extract has been reported. The prepared IONPs were characterized with fourier-transform infrared spectroscopy (FT-IR), ultraviolet-visible spectroscopy (UV-VIS), zeta potential, scanning electron microscopy (SEM), and energy-dispersive X-ray spectroscopy (EDX). The cytotoxic effects of IONPs on MCF-7 breast cancer cell line were studied by MTT assay, and migrative effect of its were carried out by the wound healing assay. It was found that the mean particle size of IONPs was 675 ± 25 nm, and the polydispersity index was 0.265 PDI. It was also determined that these nanoparticles had an anti-proliferative impact on the MCF-7 breast cancer cell line depending on the dosage. Characterization results support the successful synthesis of nanoparticles, and the dose-dependent cytotoxic effects of nanoparticles on MCF-7 cells also make it a potential chemotherapeutic agent for breast cancer treatment.

## 1. Introduction

Nanotechnology is the one of up-to-date workspaces, which is aiming to develop materials with a range of 1–100 nm dimensions. With the development of nano-scale production, metal oxides like titanium dioxide (TiO_2_), silver oxide (AgO), gold oxide (AuO), copper oxide (CuO), iron oxide (FeO), zinc oxide (ZnO) have found wide application areas [1–3]. In biomedicine, iron oxide nanoparticles (IONPs) have various applications such as magnetic resonance imaging [4], isolation of proteins and DNA [5,6], drug delivery systems [7], cytotoxicity and anti-microbial studies [8–10]. Physical, chemical, and green synthesis methods are used extensively for the production of metal oxide nanoparticles. Physical methods require expensive material and equipment, high pressure and excessive temperature. In the chemical methods, toxic chemicals such as sodium borohydride and hydrazine hydrate are used as reducing agents, which can cause severe harm to the nature and to the alive [11–13]. On account of these disadvantages of chemical and physical methods, green synthesis is proposed to be an appropriate alternative to these methods. Bacteria, algae, fungi and plants are frequently preferred in the green synthesis of nanoparticles. Among these, plant extracts are more favorable because it decreases the hazard of more contamination by reducing the reaction time and preserving the cell structure. Medicinal plant extracts are a significant and generous source of bioactive compounds such as amino acids, proteins/enzymes, polysaccharides, fatty acids, and polyphenols that can perform cytotoxic activity in different cancer cell lines. On the other hand, these bioactive compounds can reduce positive charged metal ions and stabilize the nanoparticles to intended sizes and shapes [14,15].

To synthesize iron nanoparticles, the extract of *Lagenaria siceraria* , *Myrtus communis* , *Rhus punjabensis* , *Aesculus hippocatanum* were carried out in the recent past [16–19]. *Brassica oleracea var. capitata sub.var. rubra* is a autumn plant that grows almost anywhere in the world [20]. It has been reported that the red cabbage aqueous extract prepared by boiling method contains phenolic compounds, flavonoids, glucosinolates, sulforaphane, ascorbic acids, and anthocyanin pigments [21–23]. Also, it contains a high percentage of anthocyanin pigments that gives the specific color to red cabbage [24]. In the previous study conducted, it was determined that iron nanoparticles were synthesized using anthocyanin-rich-red cabbage extract and had an antimicrobial effect. It has been emphasized that the anthocyanins in the structure of red cabbage stabilize the structure of Fe_3_O_4 _nanoparticles and increase their antimicrobial activity [10]. These metabolites are thought to facilitate the synthesis and stabilization of iron oxide nanoparticles. In the light of this information, iron oxide nanoparticles (IONPs) were synthesized using *Brassica oleracea var. capitata sub.var. rubra* peel extract, and their cytotoxic and wound closure effects were investigated on MCF-7 breast cancer cell lines. 

## 2. Materials and method

### 2.1. The preparation of Brassica oleracea var. capitata sub.var. rubra peel extract

*Brassica oleracea var. capitata sub.var. rubra,* which weighs approximately 700 grams, harvested in the Aydın region were purchased from the marketplace in February 2020. Localization of market was 37°51′06.7″N 27°48′33″E. *Brassica oleracea var. capitata sub.var. rubra* was peeled off. Then, gathered peels were washed 3 times with distilled water. Peels were chopped with a food processor. A total of 100 g of *Brassica oleracea var. capitata sub.var. rubra* peels and 200 mL distilled water were put into an erlenmeyer. This mixture was warmed up with a magnetic stirrer for 2 h at 100 °C. Finally, the mixture was filtered with Whatman filter paper (Grade1), and the filtrate was kept at +4 ^o^C until it was used in nanoparticle synthesis. [25].

### 2.2. The synthesis of IONPs

**Figure 1:**
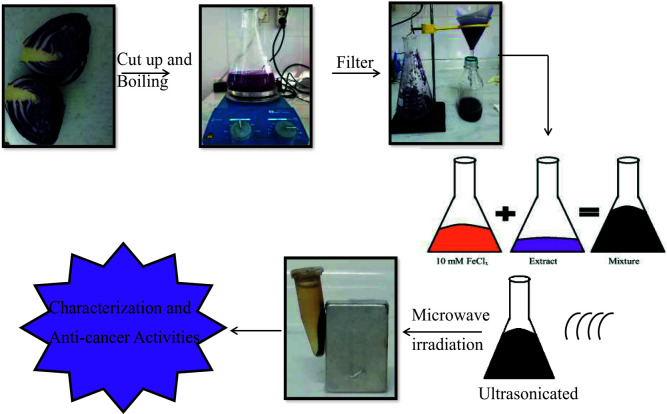
Illustration of IONPs synthesis.

20 mL of 10 mM iron (III) chloride solution and 10 mL of *Brassica oleracea var. capitata sub.var. rubra* peel extract was added in erlenmeyer. This mixture was ultrasonicated for 30 min. Then, it was exposed to 360 W microwave irradiation for 5 min. After the mixture was cooled to room temperature, the formed magnetic nanoparticles were attracted with a magnet, and the supernatant was discharged. The magnetic pellet was respectively washed five times with water and ethanol to eliminate organic residuals. After all, the pulverizing form of nanoparticles was gotten by drying in a 60 °C oven [26,27]. Illustration of IONPs synthesis with *Brassica oleracea var. capitata sub.var. rubra* peel extract was shown in Figure 1. 

### 2.3. Characterization of IONPs

The ultraviolet-visible (UV-Vis) spectrum of iron nanoparticles was taken by spectrophotometer (Thermo Scientific Multiscan Spectrum 1500) with a range of 200–800 nm. The functional group analysis of IONPs was performed taking measurements in the range of 400–4000 cm^–1^ with Fourier-transform infrared (FT-IR) spectrophotometer (Shimadzu IR 8000). Structural properties and surface morphological properties of IONPs were determined by scanning electron microscopy (SEM) (Zeiss LEO 1430 VP). Elemental composition analyses of IONPs were analyzed by energy-dispersive X-Ray (EDX) spectroscopy (LEO 1430 VP). Zeta potentials, particle distribution, and size of nanoparticles were determined using Zeta Sizer-Nano ZS (Malvern Instruments, England). Before the zeta measurement, nanoparticles were diluted with pure water and sonicated for 10 min.

### 2.4. Cell culture

MCF-7 breast cancer cells were grown and maintained in a 75 cm^2^ flask with Dulbecco’s Modified Eagle Medium (DMEM) medium including 10% FBS, 100 µg/mL streptomycin, and 100 U/mL^−1^ penicillin. The cultures were incubated with a humidified atmosphere, including 5% CO_2_, at 37 °C. Culture medium was re-added with fresh medium every two days until catch up a proper confluency of about 90%. All medium and cell culture reagents were purchased from Gibco Company. Cell studies were repeated multiple times.

### 2.5. Antiproliferative assay

The effect of IONPs on breast cancer cell viability was determined using a based-on tetrazolium salt formation assay with MTT dyes. Shortly, the MCF-7 cells were seeded into a 96-well plate at a volume of 1×10^4^ cells/well. Cells were incubated for 24 h to adhere to the bottom of the wells. IONPs at different doses (1–1000 μg/mL) were added onto the cells and incubated during 24 h. Then, the culture medium in the wells was ejected, and the wells were washed with PBS three times. After fresh medium added to all wells, 10 μL from MTT dye (0.5 mg/mL) were added to each well. The cells were kept in incubation for 4 h at 37°C. A total of 100 μL DMSO was added per well to dissolve the formed formazan dye after discharging all the culture medium. The absorbance of each well was measured on a microplate reader (Biotek Co., USA) at the wavelength of 570 nm [28]. The % cell viability was calculated using the formula given below in equation (1).

% Cell viability = (OD test sample)/ (OD control) × 100 (Eq. 1)

### 2.6. Wound healing assay

Cells were seeded into a 12 well culture plate at a volume of 1×10^5^ cells/well. For the achieve confluence at the bottom of wells, cells were incubated overnight in DMEM medium. IONPs (100 and 1000 μg/mL) were added onto the cells and incubated for 24h. To modeling the wound formation, each well was drawn with the pipette tip in its mid-point. All wells were washed with PBS twice to suspend floating and non-adhering cells. Then, the fresh DMEM medium was added to each well. Subsequently, during 24 h, the cells migrated into the drawn zone were taken photography under an inverted microscope. The amount of 24-h wound closure relative to the onset time was calculated using the Image J program [29]. 

### 2.7. Apoptosis assay

The effect of IONPs on breast cancer cell apoptosis was determined with Annexin V & Dead Cell Kit using Muse cell analyzer. Cells were seeded into 6 well culture plates and treated with IONPs (100 and 1000 μg/mL) for 24h. After the incubation, the medium was emptied, and the cells were washed by PBS twice. Cells were removed using trypsin-EDTA and suspended with DMEM. Quickly, 100 μL cell suspension and 100 μL Annexin-V reagent were suspended and incubated for 20 min in the dark at room temperature. After, cells were measured for apoptosis and live cell count. 

## 3. Results and discussion

### 3.1. Synthesized and characterization of synthesized IONPs

In recent years, plant extracts are more preferred in nanoparticle synthesis due to their inexpensive and environmentally friendly nature. When looking at the biosynthesis using red cabbage extract with different metal ions, copper [30], silver [31], and iron nanoparticles [10] stand out. Previous studies focused on the antioxidant and antimicrobial effects of these biosynthesis using aqueous red-cabbage extract. In this study, the anti-cancer effects of iron nanoparticles synthesized from red cabbage aqueous extract were investigated for the first time.

**Figure 2:**
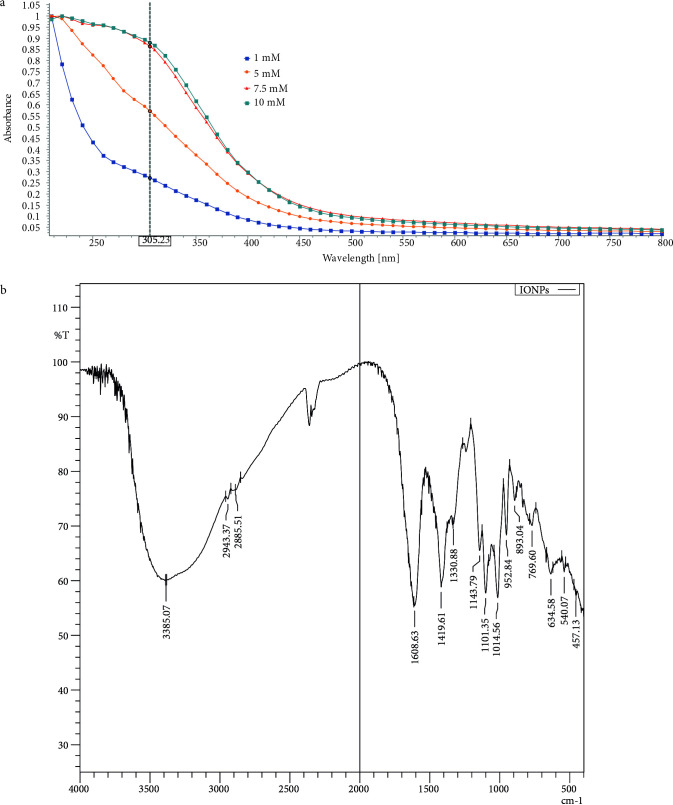
Characterization of synthesized IONPs. A) The UV-Vis spectrum of IONPs. B) The FTIR spectrum of IONPs.

The altering color of the reaction environment (visual observation) throughout the reaction time is the prime sign of nanoparticle synthesis. This color altering emerges owing to the excitation of the surface plasmon on metal nanoparticles. To determine the effect of FeCl_3 _concentration on nanoparticle synthesis, concentration of *Brassica oleracea var. capitata sub.var. rubra* extract was fixed for reaction. Nanoparticle synthesis was performed at 10 mM FeCl_3_ since it gave the maximum reaction yield. After the leaf extract of *Brassica oleracea var. capitata sub.var. rubra* was added to the 10 mM ferric (II) sulfate solution, the color changed to dark black. The absorption spectrum 1 μg/mL, 5 μg/mL, 7.5 μg/mL and 10 μg/mL of IONPs spanned a wide range from 300 to 500 nm (Figure 2A). This band indicates the formation of IONPs because it is within the range of the surface plasmon resonance (SPR) for IONPs [32,33].

FTIR analysis is frequently preferred to identify the existence of functional groups on iron nanoparticles. The FTIR spectrum of IONPs show the prominent peaks at the 3358, 1608, 1419, 1330, 634, 576 and 457 cm^–1^ wavenumbers (Figure 2B). The broad peak at 3385 cm^–1^ is featured to hydrogen-bonded O-H stretching. The sharp peak at 1608 cm^–1^ is assigned to C=O stretching vibrations. The sharp peaks of 1419 and 1330 cm^–1^ are based on H-C-H vibration bending. Fe-O-Fe stretching vibrations were monitored at 634, 540 and 457 cm^–1 ^[34–36].

**Figure 3:**
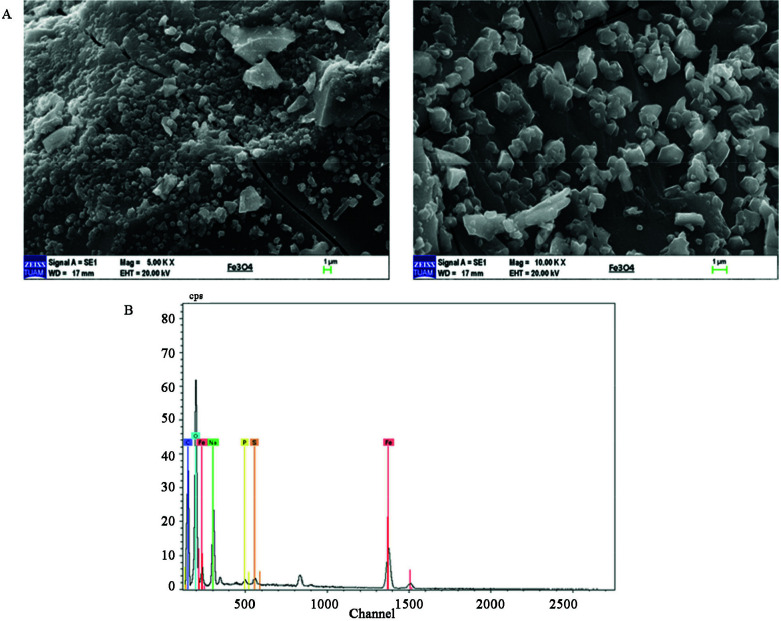
A) The SEM image of IONPs. B) Energy-dispersive X-ray spectroscopy (EDX) spectrum of IONPs

Scanning electron microscopy technic has been utilized to determine the surface size and morphology of the IONPs [37–39]. Figure 3A shows the morphology of the iron nanoparticles synthesized by *Brassica oleracea var. capitata sub.var. rubra* aqueous extract. Agglomeration of IONPS may be caused owing to the swift loss of intermittent solvent [40]. Six hundred nm and 327 nm sized particles can be seen easily after 10000 times enlargement. The elemental scheme of IONPs was calculated by using SEM accoutered with an energy dispersive X-Ray detector. EDX spectrum is shown the presence of iron nanoparticles as 12.94 % (Figure 3B). The other elements such as carbon, phosphorus, sulphur and sodium are probably caused by *Brassica oleracea var. capitata sub.var. rubra* residues on the iron nanoparticles [41,42]. Additionally, these elements exist naturally in the combination of red cabbage that also indicates the origin of growth [43]. 

**Figure 4:**
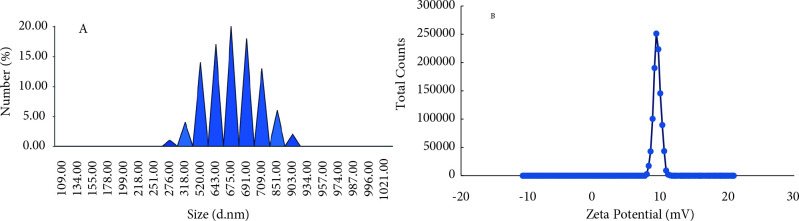
A) Size distrubition and B) Zeta potential of IONPs.

Nanoparticles used for chemotherapy need to be absorbable to cell membranes. Some studies proposed that the size of nanoparticles exhibit a critical act in their fusion and passing to cell membranes. In general, particles with a particle size between 100–200 nm are usually taken into the cell by receptor-mediated endocytosis, whereas greater particles must be removed by phagocytosis [44,45]. In the case of evaluating the size of the iron nanoparticles synthesized by *Brassica oleracea var. capitata sub.var. rubra* leaf extract, it was obtained that the average particle size was 675 ± 25 nm, and the polydispersity index was 0.265 PDI (Figure 4A). According to these results, IONPs are probably taken into phagocytosis into MCF-7 cells.

To understand how nanoparticles will behave in aqueous systems, the measurement of zeta potential or surface charge potential is a valuable data. The theoretical limit of stability of nanoparticles in a solvent system is ~30 mV [46,47]. Zeta potential value of IONPs synthesized by *Brassica oleracea var. capitata sub.var. rubra* leaf extract determined as 9.59 mV (Figure 4B). This result clearly shows that IONPs synthesized by us are within the desired limits for stability. 

### 3.2. Cell viability of MCF-7 cells treated with IONPS

**Figure 5:**
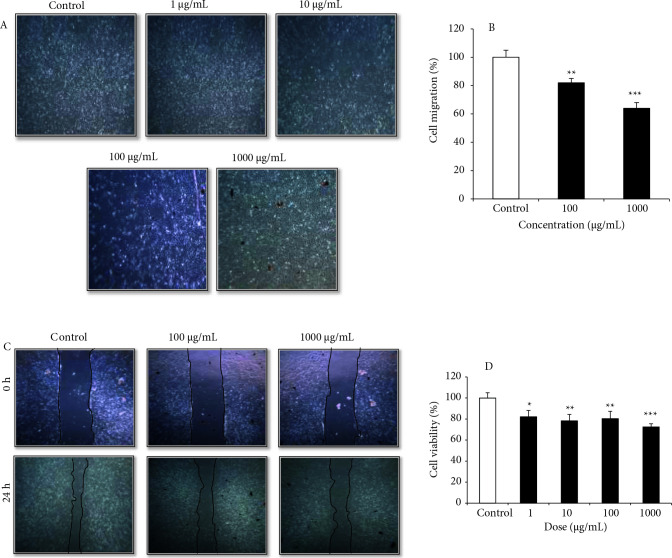
The effect of IONPs in MCF-7 breast cancer cells. A) Cell morphological changes after treatment with 1–1000 μg/mL doses of IONPs in MCF-7 cell lines for 24 h. B) Graphical illustration of % cell survival rate of MCF-7 cells after treatment with 1–1000 μg/mL doses of IONPs. C) Scratch wound assay images of MCF-7 cells treated with 100–1000 μg/mL IONPs. D) The graphical illustration of wound closure area of MCF-7 cells after treatment with 100–1000 μg/mL IONPs (*p < 0.05, **p < 0.01, ***p < 0.001 compared to control cells).

Many studies have proved that plant extracts’ bio components have cytotoxic activity against various cancerous and normal cell lines [48–50]. These bioactive components can pass to the surface of nanoparticles during the green synthesis process. Therefore, metal nanoparticles are frequently used in anticancer studies. Treatment of MCF-7 cells with 1–1000 µg/mL doses of IONPs synthesized with *Brassica oleracea var. capitata sub.var. rubra* aqueous extract inhibited the proliferation of the cells. When morphological images of the MCF-7 cells are examined (Figure 5A), it is observed that the cells treated with especially 100–1000 µg/mL IONPs show signs of apoptosis, such as blebbing the plasma membrane and shrinkage of the cell. Also, at these doses in the range of 100–1000 µg/mL, the cells move away from each other and float in the medium by rising from the well surface [51,52]. The results of MTT assay demonstrated that MCF-7 cells exposed to IONPs for 24 h resulted in concentration-dependent cytotoxicity. With increasing concentration of IONPs (1,10,100, 1000 µg/mL), the percentage viability was decreased from 100% to approximately 72.5% (Figure 5B). The results were supported by a study that iron nanoparticles synthesized with *Allium saracilum* extract could perform cytotoxic effect on HeLa and MCF-7 cells above 250 µg/mL and 500 µg/mL doses, respectively [53]. In another study, Zangeneh et al. stated that the 592 µg/mL dose of iron nanoparticles synthesized with *Falcaria vulgaris* extract reduced the cell viability of HUVEC cells to 50% [54]. Compared to this study, it appears that the iron nanoparticles synthesized with *Brassica oleracea var. capitata sub.var. rubra* leaf extract are more cytotoxic. Increased cytotoxic effect in our study is probably due to bioactive compounds from the *Brassica oleracea var. capitata sub.var. rubra* extract on the iron nanoparticle. 

### 3.3. The effect of wound healing on MCF-7 cells treated with IONPS

Cancer cells have the ability to form colonies on their own and grow rapidly and form tumors in the tissue where they grow. Wound healing assay that measures of motility of cells is often used to determine the adhesion ability and metastatic potential of cancer cells. This method is constructed on observation of cell migration into a “wound” that is created on a cell monolayer [55]. Figure 5C shows the formed wounds at 0 h and the healing progression at 24 h in MKN-45 cells. As shown in Figure 5D, cellular migration was inhibited by up to 87% and 27% at 24h with 100 µg/mL and 1000 µg/mL IONPs, respectively. 

### 3.4. The effect of apoptosis on MCF-7 cells treated with IONPS

**Figure 6:**
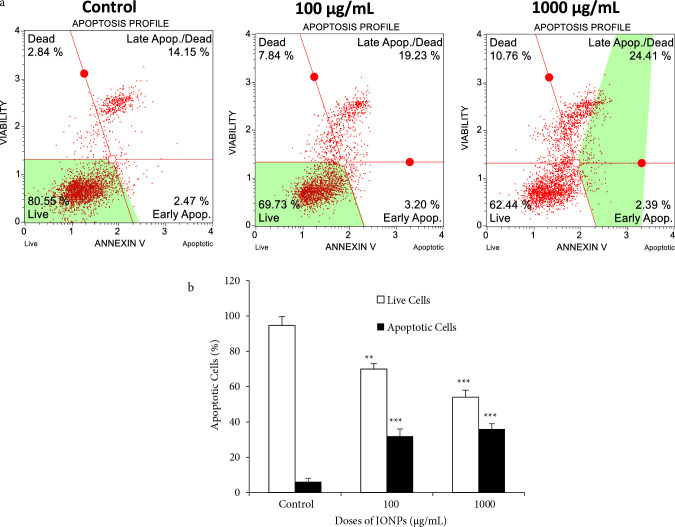
The apoptotic effect of IONPs in MCF-7 breast cancer cells. A) The scatter plot graphics of cells after treatment with 100 μg/mL and 1000 μg/mL doses of IONPs in MCF-7 cell lines for 24 h. B) The percentage of live and total apoptotic cells (early and late) of MCF-7 cells after treatment with 100 μg/mL and 1000 μg/mL doses of IONPs (**p < 0.01, ***p < 0.001 compared to control cells).

Measuring the percentage of cells leading to apoptosis is an important parameter in evaluating the cell health [56]. To investigate cells undergoing apoptosis, we carried out analysis with Annexin V assay. In cells undergoing apoptosis, the cell membrane asymmetry is lost, and phosphatidylserine is exposed on the membrane surface. In this case, phosphatidylserine and annexin-V binding are increased, so the number of apoptotic cells is detected [57]. After treated with 100 µg/mL and 1000 µg/mL IONPs, the percentage of total apoptotic MCF-7 cells respectively increased to 22.43% and 26.8% compared with control cells (Figure 6A). It was observed that the percentage of living cells decreased significantly in MCF-7 cells as a result of treatment with IONPs (Figure 6B, p < 0.001). Marycz et al. reported similar results that 4B12 osteoclast cells treated with iron nanoparticles had a total apoptotic cell ratio of 29.75% [58]. It has been previously shown that hematite (α-Fe_2_O_3_) synthesized chemically without biosynthesis induces apoptosis in MCF-7 breast cancer cells in iron nanoparticles [59]. Many studies showed that metal nanoparticles enter the cell and induce oxidative stress, causing downregulation of anti-apoptotic proteins and triggering apoptosis in cancer cells by disrupting the plasma membrane structure [60].

## 4. Conclusion

IONPs are widely used in nanotechnology, biotechnology, and medical fields. In recent years, researchers have focused on the development of cost and time-effective methods for the synthesis of IONPs. In this study, which was performed with green synthesis, IONPs were synthesized using *Brassica oleracea var. capitata sub.var. rubra* leaf extract. In this way, both the physical methods that require expensive equipment are avoided and the chemical methods using chemicals that are harmful to the nature and living things. In addition, due to the cytotoxic effect of iron nanoparticles synthesized with *Brassica oleracea var. capitata sub.var. rubra* on MCF-7, cells may have the potential to be used as a chemotherapeutic agent in the cure of breast cancer.
